# Hyper-active gap filling

**DOI:** 10.3389/fpsyg.2015.00384

**Published:** 2015-04-10

**Authors:** Akira Omaki, Ellen F. Lau, Imogen Davidson White, Myles L. Dakan, Aaron Apple, Colin Phillips

**Affiliations:** ^1^Department of Cognitive Science, Johns Hopkins UniversityBaltimore, MD, USA; ^2^Department of Linguistics, University of Maryland, College ParkMD, USA

**Keywords:** filler-gap dependency, active gap filling, prediction, verb transitivity, island, plausibility mismatch effects, eye-tracking

## Abstract

Much work has demonstrated that speakers of verb-final languages are able to construct rich syntactic representations in advance of verb information. This may reflect general architectural properties of the language processor, or it may only reflect a language-specific adaptation to the demands of verb-finality. The present study addresses this issue by examining whether speakers of a verb-medial language (English) wait to consult verb transitivity information before constructing filler-gap dependencies, where internal arguments are fronted and hence precede the verb. This configuration makes it possible to investigate whether the parser actively makes representational commitments on the gap position before verb transitivity information becomes available. A key prediction of the view that rich pre-verbal structure building is a general architectural property is that speakers of verb-medial languages should predictively construct dependencies in advance of verb transitivity information, and therefore that disruption should be observed when the verb has intransitive subcategorization frames that are incompatible with the predicted structure. In three reading experiments (self-paced and eye-tracking) that manipulated verb transitivity, we found evidence for reading disruption when the verb was intransitive, although no such reading difficulty was observed when the critical verb was embedded inside a syntactic island structure, which blocks filler-gap dependency completion. These results are consistent with the hypothesis that in English, as in verb-final languages, information from preverbal noun phrases is sufficient to trigger active dependency completion without having access to verb transitivity information.

## Introduction

A leading goal of sentence processing research is to understand how the parser adapts to a multitude of linguistic differences across languages to enable successful comprehension. In this regard, comparisons of verb-medial and verb-final languages have provided a valuable source of evidence (Mazuka and Lust, 1990; Inoue and Fodor, 1995). The main verb contains rich information such as subcategorization and thematic role information that is critical for constructing structural analyses and interpretations (e.g., Chomsky, 1965; Grimshaw, 1990; Pollard and Sag, 1994; Levin and Rappaport Hovav, 1995). Much experimental evidence shows that the verb is a valuable source of information for parsing (e.g., Ford et al., 1982; Tanenhaus and Carlson, 1989; Boland et al., 1990; MacDonald et al., 1994; Spivey-Knowlton and Sedivy, 1995; Garnsey et al., 1997; Mauner and Koenig, 2000; Traxler et al., 2002; Blodgett and Boland, 2004; Snedeker and Trueswell, 2004). The importance of the information from the verb head has engendered theoretical claims that structure building processes do not even start until the parser encounters the head of a phrase (e.g., verbal head) to be constructed, even in verb-final languages where this would be significantly delayed (Abney, 1989; Pritchett, 1992).

However, subsequent empirical research on verb-final languages like Japanese or German has generated evidence against such head-driven parsing theories in their strongest form, demonstrating that the parser uses various morphological and syntactic cues to incrementally build structures and interpretations in verb-final languages (Bader and Lasser, 1994; Koh, 1997; Clahsen and Featherston, 1999; Kamide and Mitchell, 1999; Konieczny, 2000; Bornkessel et al., 2002; Felser et al., 2003; Kamide et al., 2003; Aoshima et al., 2009; Yoshida, unpublished doctoral dissertation). Thus, although verb information strongly influences parsing decisions when available, speakers of verb-final languages often begin building syntactic and semantic structure in advance of the verb.

These findings raise the question of whether pre-verbal structure building reflects a language-specific adaptation to the processing demands of verb-finality, or rather a property of a general parsing architecture that speakers of all languages use. For example, consider less frequent cases in verb-medial languages where multiple arguments precede the verb. A classic example of this comes from processing of ‘filler-gap’ dependencies as illustrated by the relative clause construction shown in (1), where the object noun phrase (NP) *the city* (called the *filler*) is dislocated from the post-verbal thematic position (called the *gap* ^1^), and the parser needs to associate the filler and the gap in order to assign a thematic interpretation.

(1) The city that the author visited ____ was named for an explorer.

It has been reported that speakers of verb-final languages complete filler-gap dependencies in advance of verb information, associating the filler with the earliest structural position where a thematic role could be assigned (pre-verbal object gap creation: Nakano et al., 2002; Aoshima et al., 2004). The current study examines whether this may also be the case in a verb-medial language like English, and whether pre-verbal gap creation is a language-general parsing procedure rather than an adaptation specific to verb-final languages. Under this hypothesis, we predict that English speakers should posit a gap irrespective of whether the verb ultimately licenses a direct object gap position, and that signs of reading disruption should be observed in cases where the verb does not accommodate a direct object.

We report the results of three on-line reading experiments in English that tested this prediction by examining the effect of verb transitivity on reading times in filler-gap configurations. The results are consistent with the hypothesis that the parser actively associates the filler with the verb in advance of the verb across languages, regardless of differences in verb positions. These results suggest that the procedure for filler-gap dependency completion may be uniform across languages, and are consistent with the view that the parser predictively constructs rich representations at the earliest possible moment in advance of critical bottom–up evidence.

### Background on Active Filler-Gap Dependency Processing

Past research on filler-gap dependency processing has established that the parser postulates a gap before there is sufficient bottom–up evidence to confirm that analysis (*Active gap filling:* Fodor, 1978; Crain and Fodor, 1985; Stowe, 1986; Frazier and Flores d’Arcais, 1989). For example, Stowe (1986) observed the so-called *Filled gap effect* in (2), i.e., slower reading times at the direct object position *us* in the wh-fronting condition (2a) than in a control condition that did not involve wh-fronting (2b). This pattern of reading times suggests that the parser had already posited a gap following the transitive verb, before checking whether the direct object position was occupied.

(2) a. My brother wanted to know who Ruth will bring us home to ____ at Christmas.
       b. My brother wanted to know if Ruth will bring us home to Mom at Christmas.

Converging evidence comes from an eye-tracking experiment by Traxler and Pickering (1996), who manipulated the thematic fit between the filler and the potential verb host, as in (3).

(3) We like the city/book that the author *wrote* unceasingly and with great dedication about _____ while waiting for a contract.

Traxler and Pickering found a plausibility mismatch effect at the critical verb in (3), i.e., the first fixation time at the optionally transitive verb *wrote* increased when the filler was an implausible object of the verb (i.e., *the city*), compared to when the filler was a plausible object of the verb (i.e., *the book*). This suggests that at least as early as the verb position, the parser postulates a gap and analyzes the filler as the object of the verb, even when the filler is a poor semantic fit to that role. In fact, there is ample time course evidence for active object gap creation, using a variety of dependent measures such as reading time and gaze duration measures (Crain and Fodor, 1985; Frazier, 1987; Frazier and Clifton, 1989; de Vincenzi, 1991; Pickering and Traxler, 2001, 2003; Aoshima et al., 2004; Phillips, 2006; Wagers and Phillips, 2009), cross-modal priming (Nicol and Swinney, 1989; Nicol, 1993; Nakano et al., 2002), visual world eye-tracking (Sussman and Sedivy, 2003) as well as event-related potentials (Garnsey et al., 1989; Featherston et al., 2000; Kaan et al., 2000; Felser et al., 2003; Phillips et al., 2005; Gouvea et al., 2010).

The work summarized above may suggest that filler-gap dependency completion is triggered only after the parser gains access to the verb and confirms that the verb is transitive and is able to syntactically accommodate an object. However, evidence that active dependency completion does not depend on verb information has been presented by studies that investigated (i) subject gap creation in English, as well as (ii) object gap creation in verb-final languages. For example, Lee (2004) used sentences like (4) to reveal a filled gap effect in the subject NP position.

(4) a. That is the laboratory which, on two different occasions, Irene used a courier to deliver the samples to ___.
       b. That is the laboratory to which, on two different occasions, Irene used a courier to deliver the samples ___.

Here, the content of the wh-filler is manipulated in such a way that the wh-filler can plausibly be a subject (4a) or not (4b). The results showed a longer reading time at the subject NP *Irene* in (4a) than in (4b), suggesting that the parser had postulated a subject gap before encountering the actual subject NP. Although this interpretation has been challenged (Staub, 2010), it would in any case not be surprising that the parser actively creates a subject gap without having access to verb information, given that a subject is present in any sentence, regardless of verb properties. In this sense, if verb information were to play a role in the parser’s attempt to posit a gap, the critical empirical evidence should come from dependency completion at the object position, where the presence or absence of an object gap relies on properties of the verb.

Evidence for pre-verbal object gap creation has been reported for verb-final languages like Japanese in which the object gap position linearly precedes the verb. For example, Aoshima et al. (2004) examined processing of scrambling sentences in which a dative object NP was dislocated to the sentence initial position, and found a filled gap effect at a pre-verbal dative object position for the first verb phrase (VP) in the sentence (see also Omaki et al., 2014). Using similar sentences, Nakano et al. (2002) reported evidence for an antecedent priming effect for the scrambled NP at a pre-verbal gap position, although the priming effect was only found in the high working memory span group. These data indicate that the parser can in principle complete filler-gap dependencies before accessing verb information.

In verb-medial languages, no such evidence for pre-verbal object gap creation has been reported to date. This may reflect a real difference between languages in processing strategy, and pre-verbal object gap creation in verb-final languages may reflect the parser’s adaptation to the demands of processing these languages. Maintaining a structurally unintegrated filler in memory has been argued to impose a burden on working memory (King and Just, 1991; Gibson, 1998; Gordon et al., 2002; Haarmann and Cameron, 2005). Alternatively, the parser may be architecturally constrained to assign a thematic interpretation to the filler as soon as possible (Pickering and Barry, 1991; Aoshima et al., 2004). On this view, the parser should prioritize integrating the filler into the first grammatically permissible structural position that can potentially receive a thematic role. Given that filler-gap dependencies are potentially unbounded, waiting for the verb before constructing the ultimate object gap position could impose a large processing burden on speakers of verb-final languages.

In verb-medial languages like English, verbs become available relatively earlier in the sentence, such that the average working memory cost of waiting for the verb would be less than in verb-final languages. The advantage of waiting for the verb information is that the parser can reduce the likelihood of making risky commitments, because the verb may turn out to be intransitive and disallow an object NP analysis for the filler. In English, therefore, the parser may create an object gap position only after the verb is confirmed to be transitive. This still constitutes active gap filling, in the respect that the ultimate gap position may turn out to be somewhere later than the object position [e.g., after a late-arriving preposition gap, as in (2) and (3)]. Let us call this a *conservative active gap filling* mechanism, since the bottom–up subcategorization information from the verb still plays a critical role in the parser’s decision on whether to postulate an object gap or not. This view of active gap filling is rather standard for explaining filler-gap dependency completion in verb medial languages like English. For example, McElree and Griffith (1998) and McElree et al. (2003) have argued that the dependency completion process is triggered when the parser accesses information from the verb and initiates the retrieval process for the filler that is stored in working memory (see also Pickering and Barry, 1991; Lewis and Vasishth, 2005).

On the other hand, pre-verbal object gap creation in verb-final languages may reflect a language-general property of the processing architecture, although evidence for such mechanisms may be simply more difficult to obtain in verb-medial languages. In the English filler-gap case, for example, in any parser that adopts some form of left-corner strategy (Kimball, 1975; Abney and Johnson, 1991; Resnik, 1992; Shieber and Johnson, 1993; Stabler, 1994; Crocker, 1996; Lewis and Vasishth, 2005; Gibson, unpublished doctoral dissertation), the presence of the subject NP allows the parser to predict the presence of a VP. Given that a VP can contain an object NP position, the parser could project a VP with an object NP slot and assign the filler to this object position before confirming whether the upcoming verb is a transitive verb or not. Let us call this a *hyper-active gap filling* mechanism, because this involves a more risky predictive structure building process than is standardly assumed for active object gap creation in English. Filler retrieval and structural integration is still integral to the hyper-active gap filling mechanism, but the crucial difference is in what information triggers retrieval and integration, and consequently, at what point in the sentence this process is executed.

It is important to note that either of these two active gap filling mechanisms is compatible with the existing data on active object gap creation reviewed above. A filled gap effect only indicates that the gap had been created before the actual object NP is processed, and this result is compatible with both accounts, given that both hyper-active gap filling and conservative active gap filling mechanisms assume that object NP gap creation happens before or on the verb. A plausibility mismatch effect indicates that when the verb is potentially transitive, then the semantic fit between the filler and the verb is immediately assessed. This is also predicted by both accounts. The assessment of the semantic relation between the filler and the verb requires the parser to access the content of the verb, by which point the object gap position should have been created on either account. Thus, neither paradigm allows us to tease apart the two hypotheses on what kind of information is sufficient for triggering object gap creation.

In the current study we aim to tease apart the predictions of two hypothesized mechanisms for active object gap creation processes. If English speakers construct the gap site before encountering the verb, just like speakers of verb-final languages, then disruption should be observed in filler-gap configurations when the verb turns out to be intransitive, relative to transitive verbs (e.g., *The party that the student arrived/planned…*). According to the conservative active gap filling mechanism outlined above, the parser waits for a transitive verb before postulating the corresponding gap structure. Here, no disruption is expected at an intransitive verb, since the parser has not postulated a gap that would require a transitive verb.

Two previous studies are relevant to the two hypotheses about active object gap creation in English. Previous work by Pickering and Traxler (2003) examined the effect of subcategorization frequency in optionally transitive verbs (e.g., *Those are the lines/props that the author spoke [about]…*). It was found that readers did not take subcategorization frequency into account in deciding where to posit a gap, as there was a strong preference to posit a gap in the verb object position (NP complement) even with verbs that more frequently take a PP complement. The absence of subcategorization frequency effect in active object gap creation could be taken to indicate that verb information is not relevant for object gap creation processes. However, all of the verbs in Pickering and Traxler’s study could grammatically accommodate an NP complement, and the parser may therefore have relied on the transitivity information of the verb to create an object gap. Therefore, this finding does not distinguish the predictions of the two proposed mechanisms for active object gap creation.

To our knowledge, the only previous test of these two active object gap creation hypotheses is in Experiment 3 of Staub (2007). The test sentences in this experiment (5a–d) manipulated the transitivity of the verb (*called* vs. *arrived*) and sentence structure (relative clause with a gap vs. simple declarative with no gap). The filler was manipulated to be an implausible object of the transitive verb (*gadget-called*). Under the hyper-active gap filling hypothesis, the parser in effect predicts the presence of a transitive verb, and therefore the reading processes in the gap conditions should be disrupted in either intransitive or transitive condition, but for different reasons: when the verb turns out to be intransitive, and processing should also be disrupted when the verb is transitive because of the plausibility mismatch effect. On the other hand, the conservative active gap filling mechanism postulates a gap only after checking whether the verb is capable of hosting an object NP, and therefore reading disruption is predicted only in the transitive gap condition due to the plausibility mismatch effect.

(5) a. The gadget that the manager *called* occasionally about…
       b. The manager *called* occasionally about the gadget …
       c. The party that the student *arrived* promptly for …
       d. The student *arrived* promptly for the party …

Staub (2007) found longer first-fixation durations in the transitive gap condition (5a) than in the transitive no-gap condition (5b), but no such difference was observed between the intransitive gap and no-gap conditions (5c) and (5d). This pattern of data supports the prediction of the conservative active gap filling hypothesis, suggesting that the parser does not create an object gap until it checks the transitivity information of the verb. One concern about this design, however, is whether the no-gap condition was truly a neutral baseline against which a transitivity mismatch could be measured, as the gap and no-gap conditions differed substantially in both the linear and structural position of the verb. As Staub (2007) points out, one piece of data suggesting that the control may not have been completely neutral is the fact that reading times on the intransitives were numerically (but non-significantly) shorter in the gap condition than in the no-gap condition. It is important to note here that the gap conditions (5a) and (5c) contain an extra NP (i.e., the head of the relative clause) prior to the critical verb region in comparison to the no-gap conditions (5b) and (5d). This may have led to a difference in the amount of contextual information available prior to the verb. Increased contextual information can facilitate processing for subsequent lexical items (Stanovich and West, 1983; Van Petten and Kutas, 1990; Kutas and Federmeier, 2000), and for this reason, lexical access for the intransitive verb in the gap condition may have become faster and masked the potential reading time slowdown associated with the structural manipulation. In an attempt to provide a better test of the predictions of the hyper-active and conservative active gap filling accounts, the current study used relative clause islands as a control condition, which allowed the target sentences to more closely match in informational content and word position.

## Experiment 1

Experiment 1 was a self-paced reading study that was designed to test the predictions of the hyper-active and conservative active gap filling hypotheses, while addressing methodological concerns about previous work. We employed the transitivity mismatch paradigm used in Staub (2007) in order to test whether a verb transitivity manipulation affects reading time at the verb. Critically, in the baseline conditions the critical verb was embedded inside a relative clause structure, a syntactic ‘island’ domain that prohibits filler-gap dependency formation (Ross, unpublished doctoral dissertation; for a review, see Szabolcsi and den Dikken, 2003). A sample set of stimuli is shown in **Table 1**.

**Table 1:** **Sample materials and conditions for Experiment 1**.

	Analysis regions										
	1	2	3	4	5	6	7	8	9	10	11
Transitive, non-island	The	city	that	the	author		wrote	regularly	about	was	named for an explorer
Transitive, island	The	city	that	the	author	who	wrote	regularly	saw	was	named for an explorer
Intransitive, non-island	The	city	that	the	author		chatted	regularly	about	was	named for an explorer
Intransitive, island	The	city	that	the	author	who	chatted	regularly	saw	was	named for an explorer
Example question	Was the city named for an explorer?					

A number of previous studies have shown that the parser respects island constraints in real-time syntactic processing, such that it avoids actively constructing filler-gap dependencies that span syntactic island boundaries (Stowe, 1986; Kluender and Kutas, 1993; McKinnon and Osterhout, 1996; Traxler and Pickering, 1996; McElree and Griffith, 1998; Wagers and Phillips, 2009; Omaki and Schulz, 2011; Yoshida, unpublished doctoral dissertation). The relative clause island condition thus provided a baseline measure of reading times for the critical transitive and intransitive verbs, independent of processes of filler-gap dependency completion. The use of island configurations allowed us to address the methodological concerns with previous work.

First, this design allowed the baseline condition to present a filler NP prior to the critical region, such that the same amount of contextual information from the lexical items was present in advance of the critical verb region across the four conditions. Second, the word position for the critical regions (Regions 7 and 8 in **Table 1**) was closely matched across conditions (word positions 6 and 7 in the non-island conditions, word positions 7 and 8 in the island conditions), and it was also placed away from the early portion of the sentence.

Furthermore, following Staub’s design, we selected transitive verbs that are implausible hosts for the filler. Under this design, the hyper-active gap filling hypothesis predicted a reading time slowdown in both the non-island transitive and the non-island intransitive conditions relative to their island counterparts, but for a different reason in the two cases. In the transitive condition, the slowdown would reflect a plausibility mismatch effect triggered by the poor semantic fit between the filler and the verb. In the intransitive condition, the slowdown would result from a transitivity mismatch effect due to the mismatch between the expected subcategorization property of the verb (i.e., transitive) and the actual subcategorization property of the verb. On the other hand, the conservative active gap filling hypothesis predicted an interaction. A reading time contrast should be observed between the non-island transitive condition and the island transitive condition due to the plausibility mismatch effect, but no corresponding contrast should be observed between the two intransitive conditions, given that the parser should not actively create an object gap in either condition. Note that the lexical difference in the critical verb region across conditions was not problematic, since the critical contrast was between non-island and island conditions within each verb type.

### Method

#### Participants

We recruited 32 native speakers of American English from the University of Maryland community. They received a course credit or were paid $10 for their participation and were naïve to the purpose of the experiment.

#### Materials

We used 28 sets of four sentences like those shown in **Table 1**. All of the stimuli from experiments reported in this paper are made available in Supplementary Materials. The transitive non-island and island conditions were taken from the implausible semantic fit conditions in Omaki and Schulz (2011), who used a modified version of the plausibility manipulation materials from Traxler and Pickering (1996). Omaki and Schulz replicated Traxler and Pickering’s plausibility mismatch effect with native and non-native speakers alike, confirming that the semantic fit between the filler and the verb affects the reading time for the verb when the verb is in a gap filling (i.e., non-island) environment, but not when the verb is inside a relative clause island. Critically, it was also found that the implausible verb-filler combination in a non-island environment (e.g., *city-wrote*) led to a significant slow down at the verb compared to its island counterpart with the same implausible verb-filler combination. Thus, even though the current experiment did not include a plausible counterpart of the implausible transitive verb condition, we could be confident that a reading time contrast between the transitive non-island and island conditions results from the semantic misfit between the filler and the verb. In other words, the finding in Omaki and Schulz’s study supports the notion that island conditions in general can be used as baseline conditions for a reading disruption associated with active object gap creation. The intransitive conditions were modeled after the transitive conditions by replacing the optionally transitive verb with unergative or unaccusative intransitive verbs (Levin and Rappaport Hovav, 1995).

The non-island and island conditions differed in the number of relative clauses. The non-island condition had only one relative clause (*the city that the author wrote/chatted regularly about*), such that the object position of the verb *wrote/chatted* was the first potential gap position after the embedded subject was encountered. In the island conditions, the critical verb was embedded inside another relative clause *the author who wrote/chatted regularly*, such that linearly this was still the first verb but grammatically the filler should not be accessible to the verb due to the relative clause island constraint. Thus, the first verb served as the critical region for testing the plausibility and transitivity mismatch effects. All the transitive verbs were optionally transitive, such that the sentences in the island conditions were all ultimately grammatical. The subcategorization frequency of the optionally transitive verbs was not controlled, since Pickering and Traxler (2003) have demonstrated that plausibility mismatch effects are attested for optionally transitive verbs regardless of subcategorization frequency. In all four conditions the same adverb immediately followed the verb, making it possible to observe potential spill-over effects. The 28 sentence sets were counter-balanced across four lists so that each participant saw only one version of the target items and consequently read seven tokens of each condition. In addition, 72 fillers of similar length and complexity were constructed and added to each list.

#### Procedure

The self-paced reading task was implemented on the Linger software developed by Doug Rohde (http://tedlab.mit.edu/‘dr/Linger/). We used a word-by-word, non-cumulative moving window presentation (Just et al., 1982). In this design, each sentence initially appears as a series of dashes, and these dashes are replaced by a word from left to right every time the participant presses the space bar. In order to ensure that the participants were paying attention while reading the sentences, all sentences were followed by yes-no comprehension questions, and feedback was provided if the questions were answered incorrectly. Comprehension questions never addressed the critical filler-gap portion of the sentence. At the beginning of the experiment, participants were instructed to read at a natural pace and to answer the questions as accurately as possible. Seven practice items preceded the self-paced reading experiment, and the order of presentation was randomized for each participant. The experiment took ∼30 min. The experiment protocol for this study was approved by the Institutional Review Board at the University of Maryland.

#### Data Analysis

The data from two items were excluded from analyses due to coding errors. Only trials in which the comprehension question was answered accurately were included in the analysis, which affected 5.7% of the trials. We also analyzed the data without excluding the trials based on comprehension accuracy, but the overall pattern of results did not change.

Self-paced reading times for the target sentences were examined for each successive region, although the words after the auxiliary *was* were combined into a single region because these lay beyond the critical regions and were unlikely to show effects relevant for the critical manipulation. The critical regions where a potential plausibility or transitivity mismatch effect was expected consist of Region 7 (i.e., the verb *wrote/chatted*) and the following Region 8 (i.e., the adverb *regularly*), in which spill-over effects could be observed. Regions 1 through 6 were predicted to show no difference across conditions, since they were lexically matched. Regions 9 through 11 could reveal reading time differences after the filler-gap dependency is completed (Region 9 hosts the true gap site), and with a possible additional difference in the island conditions due to the structural complexity associated with the extra relative clause in these conditions.

Reading time data that exceeded three standard deviations from the group mean at each region and in each condition were excluded, affecting 1.7% of the data. The remaining reading time data were analyzed using linear mixed effects models (Baayen et al., 2008). These analyses were conducted in the R environment (R Development Core Team, 2011), using the lme4 package for R (Bates et al., 2014). The fixed effects of island structure type (non-island vs. island) and verb transitivity (transitive vs. intransitive) were coded using sum contrasts, with one level of the factor coded as -0.5, and the other as 0.5. This sum contrast coding makes the mixed effect model estimates roughly comparable to the actual average reading time contrasts. The model included random intercepts for participants and items. For random slopes, we used the following procedure to determine the optimal random effect structure (for discussions: Jaeger, 2011; Barr et al., 2013). First, we constructed a fully crossed model that included the fixed effects and an interaction term as random slopes for both participants and items. This fully specified model failed to converge, plausibly due to the complexity of the model and missing data points in some of the trials (Barr et al., 2013). Next, we simplified the random effect structure by only keeping the verb transitivity factor as a random slope for participants and items. In our experimental design, the island structure is invariant across all items, and it is also known to be robust across individuals, regardless of working memory capacity (see Sprouse et al., 2012). On the other hand, the verbs differed across items, and it is possible that the subcategorization bias differs across participants. This mixed effects model converged for all regions. We computed *p* values for linear mixed effects models using the lmerTest R package (Kuznetsova et al., 2014).

#### Results

##### Comprehension accuracy

The mean comprehension question accuracy for experimental items across participants and items was 93.0%. For the non-island conditions, the transitive items were answered with an accuracy of 93.7% (SE = 1.9), and the intransitive items with an accuracy of 94.6% (SE = 1.4). For the island conditions, the transitive items were answered with an accuracy of 91.5% (SE = 1.7), and the intransitive items with an accuracy of 92.0% (SE = 2.2). The mean accuracy did not differ reliably across conditions, although the fact that the mean accuracy for island conditions was numerically lower may reflect the complexity difference between non-island and island conditions.

##### Reading time data

**FIGURE 1:**
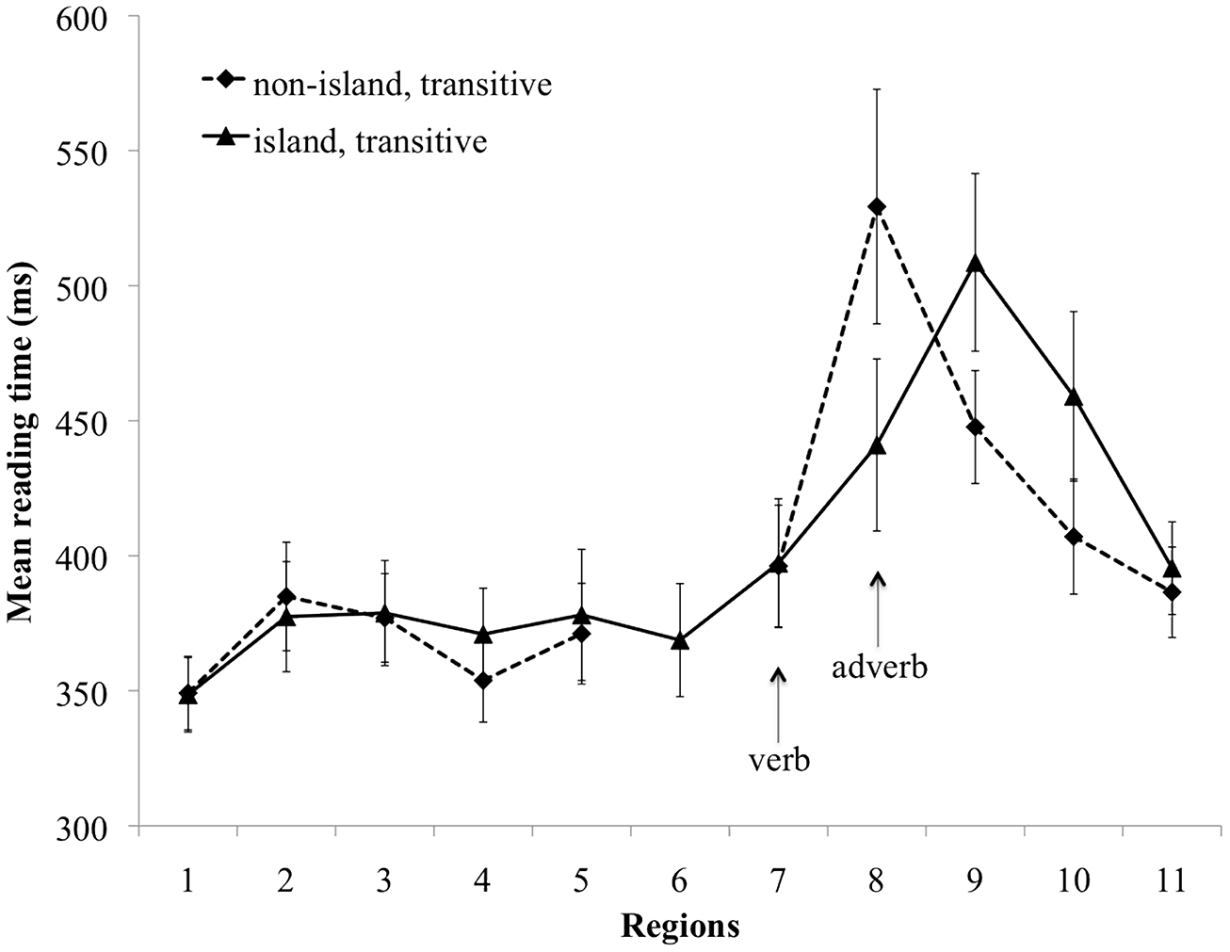
Mean reading time (ms) for the transitive non-island and island conditions. Error bars indicate standard error of the mean.

**FIGURE 2:**
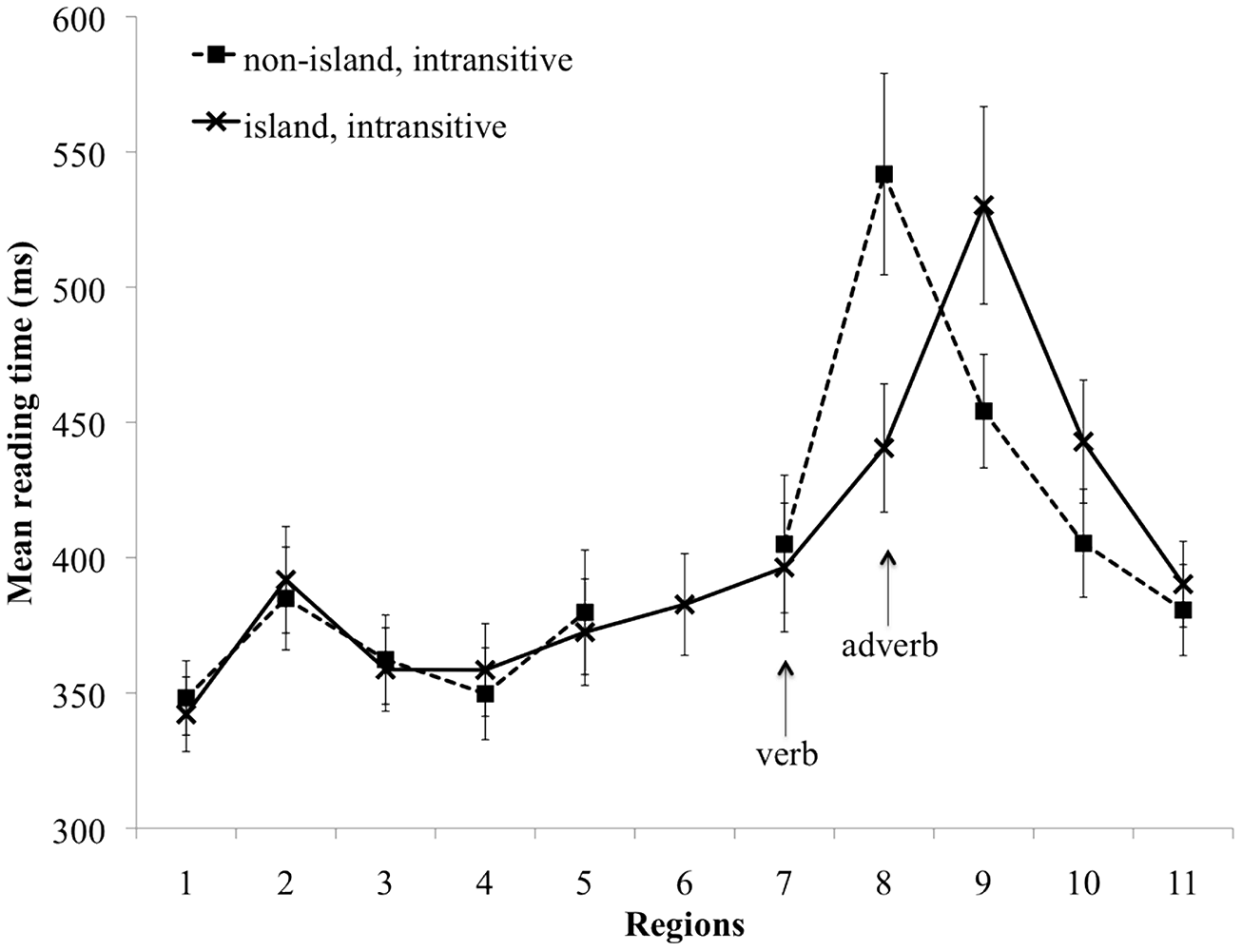
Mean reading time (ms) for the intransitive non-island and island conditions. Error bars indicate standard error of the mean.

The region-by-region mean reading time for the transitive conditions is presented in **Figure 1**, and the mean region-by-region reading time for the intransitive conditions is presented in **Figure 2**.

In the non-critical Regions 1–6, there were no significant differences in Regions 1, 2, 4–6 (*p*s > 0.06). In Region 3 there was a main effect of verb type (Estimate = -17.3, SE = 7.6, *t* = -2.27, *p* < 0.05), due to slower reading times in the transitive conditions than in the intransitive conditions (381 vs. 358 ms). Since this region was lexically matched across conditions, we conclude that this is a spurious effect. But given that the effect was small and occurred well ahead of the critical regions, this unexpected effect was unlikely to have impacted the observations in the critical regions.

At the critical verb in Region 7 there were no significant differences (*p*s > 0.1). The following spill-over region (Region 8) revealed no main effect of verb type, but there was a main effect of structure type (Estimate = -92.0, SE = 16.4, *t* = -5.61, *p* < 0.001), reflecting the fact that the non-island conditions produced significantly slower reading times than the island conditions (529 vs. 435 ms). There was no significant interaction of verb type and structure type (*p* > 0.1).

Region 9 consisted of a second verb in the island conditions and a preposition in the non-island conditions. We observed a main effect of structure type in Region 9 (Estimate = 63.7, SE = 15.9, *t* = 4.01, *p* < 0.001), as well as in Region 10 (Estimate = 46.1, SE = 11.5, *t* = 4.0, *p* < 0.001), in these cases due to slower reading times in the island conditions (Region 9: 519 vs. 451 ms, Region 10: 451 vs. 406 ms). Region 11 revealed no significant differences (*p*s > 0.09).

##### Discussion

In Experiment 1, we tested the predictions of two hypotheses about active object gap creation. The hyper-active gap filling hypothesis predicted the presence of reading disruption at intransitive verbs, because encountering an intransitive verb in a filler-gap context would be incompatible with the object gap structure constructed earlier. On the other hand, the conservative active gap filling hypothesis predicted no such reading disruption, because the parser should first consult the transitivity information of the verb to decide whether to posit an object gap or not. As a baseline for estimating the degree of disruption at the verb, we used relative clause island constructions, which block the association of the filler with the critical verb. The results were consistent with the predictions of the hyper-active account: in the region following the verb, we observed slower reading times for intransitive verbs in non-island conditions than in corresponding island conditions.

Previous work has shown a filler-gap plausibility mismatch effect at the verb such that mismatched transitive verbs in a non-island environment elicit longer reading times than their plausible non-island or plausible/implausible island counterparts (Traxler and Pickering, 1996; Omaki and Schulz, 2011), and here we replicated this finding. This effect can be interpreted as the result of active association of the filler with the transitive verb, which in these stimuli resulted in a verb–object plausibility mismatch. On the other hand, the slowdown observed in the intransitive non-island condition relative to the intransitive island condition can be interpreted as a *transitivity* mismatch. This suggests that the parser does not wait for bottom–up evidence from the verb that the verb can syntactically license a gap, but rather attempts to construct the dependency before this information is available. This slowdown cannot reflect the cost of maintaining the filler in working memory, because a filler is also being maintained at this position in the baseline island condition.

It is also important to note that the shorter reading times in the critical regions of the island conditions are theoretically informative. These findings suggest that the reading time increase in the non-island conditions is specifically due to an expectation violation following premature gap creation. A plausible alternative explanation of the reading disruption in the non-island conditions is that it reflects a more general cost associated with delaying gap creation decisions. Under this alternative account, we should expect to observe reading disruption in the island conditions as well, because gap creation must wait until the verb that follows the relative clause island region (e.g., *saw* in Region 9). However, this prediction is not supported by the data, as the reading time in the adverb region (Region 8) of the island conditions was reliably shorter than in non-island conditions.

In Regions 9 and 10, the island conditions were read more slowly for both levels of verb type. Region 9 corresponds to the word that licensed the true gap site across all conditions, and hence this slowdown could reflect a difference in the so-called integration cost (Gibson, 1998, 2000) between non-island and island conditions. Previous work on filler-gap dependency processing has demonstrated that increased complexity and length differences result in increased processing difficulties at the gap site, as measured by reading time (Gibson and Warren, 2004; Wagers and Phillips, 2014) and reduced accuracy in speeded acceptability judgment tasks (McElree et al., 2003). However, the reading time difference in Region 9 may simply be due to lexical differences (prepositions in the non-island conditions vs. verbs in the island conditions), so the reading time contrast between the island and non-island conditions may not reflect an integration cost difference.

Note that it is unlikely that the reading time contrast between non-island and island conditions in Region 8 is related to the overall complexity of the constructions used in our stimuli, given that on all accounts that we are aware of, island domains have been argued to be syntactically more complex and more taxing for working memory resources (Deane, 1991; Kluender and Kutas, 1993; Kluender, 1998, 2004; Hofmeister and Sag, 2010). The fact that the putatively less complex non-island conditions were read more slowly allows us to attribute the slowdown to processes that uniquely occur in the non-island conditions, namely filler-verb association.

In summary, the presence of both a plausibility mismatch effect and a transitivity mismatch effect lends support to the hyper-active gap filling hypothesis, and argues against a conservative active gap filling hypothesis under which transitivity information is consulted before attempting to create an object gap. This finding directly contrasts with that of Staub (2007), who did not find evidence for a transitivity mismatch effect.

However, this conclusion is not warranted until two methodological concerns are addressed. First, the design in Experiment 1 was modeled after Staub (2007), who used a plausibility mismatch design for transitive verb conditions, and transitivity mismatch design for intransitive verb conditions. Our findings differed from Staub’s as we found mismatch effects for both transitive and intransitive non-island conditions, but it is possible that some nuisance factor common to both non-island conditions led to a slow-down across the board. Stronger evidence for the hyper-active gap filling hypothesis can be obtained if we replicate the transitivity mismatch slowdown in the intransitive non-island condition, while at the same time observing no reading disruption in the transitive non-island condition. Experiment 2 accomplished this by making the filler and the verb semantically fit in the transitive conditions. The absence of reading disruption in the transitive conditions would suggest that the disruption in the non-island, intransitive condition is due to the intransitivity of the verb.

Second, it is important to note that our evidence for reading disruption for transitive and intransitive verbs (i.e., the slowdown in non-island conditions compared to island conditions) was not observed until the spill-over adverb region. Spill-over effects are widely observed in self-paced reading experiments, and it is thus common to attribute spill-over effects to processes triggered in a preceding region. However, in our experiment there is an alternative explanation for the effect in the adverb region that would not require hyper-active gap filling. For the intransitive condition, the slowdown in the adverb region could indicate that the parser had expected the presence of a preposition, which would allow structural integration of the filler. Under this alternative account, the slowdown is not due to a transitivity mismatch on the verb, but rather to a word category expectation mismatch in the adverb region that was triggered by the verb itself. This account is consistent with the conservative active gap filling hypothesis, since the parser’s expectation regarding filler-gap dependency completion is based on the information from the verb. Incidentally, the reading disruption observed in the transitive conditions of Staub (2007) was at the verb region. One possible reason for this discrepancy is the difference in the dependent measure: Staub (2007) used an eye-tracking during reading method while we used self-paced reading in Experiment 1. An eye-tracking during reading method generally provides better temporal precision than the self-paced reading method (Rayner, 1998; Rayner and Pollatsek, 2006). Thus, an eye-tracking replication of Experiment 1 may yield a transitivity mismatch effect on the verb region, and provide stronger evidence for the hyper-active gap filling hypothesis. This is addressed in Experiment 2.

### Experiment 2

Experiment 2 addressed two methodological concerns raised in Experiment 1 by removing sources of slowdown in the transitive conditions, and also by using the eye-tracking during reading method.

#### Method

##### Participants

We recruited 33 native speakers of American English from the Johns Hopkins University community, but data from one participant were removed due to calibration errors. Participants received course credit or $10 for their participation. They were all naïve to the purpose of the experiment.

##### Materials

We used 28 sets of four sentences as shown in **Table 2**. This experiment used the same transitivity mismatch logic as Experiment 1 and manipulated the verb transitivity type (intransitive vs. transitive). However, in this experiment the semantic fit between the filler and the transitive verb was always plausible, such that no reading disruption was expected at the transitive verb in the non-island condition. As in Experiment 1 we manipulated structure type (non-island vs. island), using conditions with relative clause island structures as baseline conditions. Relative clause islands provide an effective baseline, since they include the same filler NP and other lexical material as the non-island condition, while preventing dependency completion at the critical verb. As in Experiment 1, the transitive verbs were optionally transitive and the true gap position occurred outside the island domain, allowing the sentence to continue grammatically.

**Table 2:** **Sample materials and conditions for Experiment 2**.

	Analysis regions				
	Sentence initial	Pre-verb	Verb	Post-verb	Sentence final
Transitive, non-island	The book that	the author	wrote	regularly	about was named for an explorer
Transitive, island	The book that	the author who	wrote	regularly	saw was named for an explorer
Intransitive, non-island	The book that	the author	chatted	regularly	about was named for an explorer
Intransitive, island	The book that	the author who	chatted	regularly	saw was named for an explorer
Example question	Was the book named for an explorer?

The 28 sentence sets were counter-balanced across four lists so that each participant saw only one version of the target items and consequently read seven tokens of each condition. In addition, 76 fillers of similar length and complexity were constructed and added to each list.

##### Procedure

An Eyelink 1000 eye-tracker (SR Research: Mississauga, ON, Canada) was used to record eye movements. The participant’s head was stabilized by a chin rest and a forehead rest. The position of the right eye only was monitored at a sampling rate of 1000 Hz. The eye-tracker display allowed a maximum of 120 characters per line, in 10 pt Monaco font. Some filler sentences were displayed on two lines, but all target sentences were displayed on one line. Stimuli were displayed on a 21.5-inch Samsung SyncMaster monitor, and participants were seated 65 cm from the computer screen. Before the experiment started, participants were seated in front of the eye-tracker and received instructions for the experiment. A calibration routine was performed at the beginning of the experiment, and the experimenter monitored the calibration accuracy throughout the session, recalibrating when necessary. The experiment started with written instruction on the display and seven practice trials. At the beginning of each trial, a black circle was displayed on the left side of the monitor, which corresponded to the location of the beginning of the sentence. The text was displayed after the participant successfully fixated on the circle. After reading each sentence, the participant pressed a button to remove the sentence display. Each sentence was followed by a yes-no comprehension question, and the participant answered the comprehension question by pressing a left or right button. Comprehension questions never addressed the critical filler-gap portion of the sentence. The entire experiment lasted ∼35 min. The experiment protocol for this study was approved by the Homewood Institutional Review Board at the Johns Hopkins University.

##### Data Analysis

Comprehension accuracy for the target trials was 90.7%, and trials in which participants answered the comprehension question incorrectly were removed from the eye movement analyses, as data from these trials may reflect inattentive reading. For the remaining data, an automatic procedure pooled short contiguous fixations. The procedure incorporated fixations of less than 80 ms into larger fixations when they occurred within one character of each other and deleted any remaining fixations of less than 80 ms, because little information can be extracted during such short fixations (Rayner and Pollatsek, 1989). Unusually long fixations greater than 800 ms were also removed, because they usually reflect tracker losses or other anomalous events. This procedure resulted in the exclusion of 4.86% of all fixations.

For the purpose of analysis of the eye movement data, the sentences were divided into five analysis regions, as shown in **Table 2**. We report eye movement data in the following three regions: (a) the pre-verb region (*the author* in non-island conditions, *the author who* in island conditions), in order to ensure that there were no unexpected reading behavior differences that might compromise the interpretation of the data from the critical region, (b) the verb region, which is the critical region where potential transitivity mismatch effects might be observed, and (c) the post-verb region, which corresponds to the post-verbal adverb and could be used to probe for potential spill-over effects. The data in the remaining regions are not reported, because reading times at these regions are not critical for distinguishing the competing hypotheses. Moreover, after the post-verb region, the lexical items were not held constant across conditions and therefore any observed differences would be difficult to interpret. The island conditions contained one extra word, i.e., the relative pronoun (e.g., *who*), which could have affected reading times in the pre-verb region as well as regression measures for subsequent regions.

Following the data analysis procedures used in Staub (2007), four reading time measures were computed for the three regions of interests: *first fixation duration, first pass time, regression path time,* and *percent regressions* (Rayner, 1998; Rayner and Pollatsek, 2006; Staub and Rayner, 2007). First fixation duration is the duration of the very first fixation in a region, regardless of whether there is a single word or multiple words in that region. This measure is often used as an index of lexical difficulty (e.g., Reichle et al., 2003) but is also informative about the earliest syntactic processes that immediately follow lexical access (e.g., Frazier and Rayner, 1982; Sturt, 2003).

The *first-pass reading time* is calculated by summing the fixations in a region between the time when the eye-gaze first enters the region from the left and the time when the eye-gaze exits the region either to the left or the right. First-pass reading times also index early lexical and syntactic processes associated with a region, but given that they consist of multiple fixations on the same region, they may also reflect slightly later processes than the first fixation measure.

*Regression path times* are the sum of fixations from the time when the eye-gaze first enters a region from the left to the time when the eye-gaze exits the region to the right. Regression path time is identical to first-pass reading time if the eye-gaze first exits the region to the right, but if the eye-gaze exits the region to the left, then regression path times are longer than the first-pass time as they include all fixations in previous regions as well as re-fixations on the region before exiting the region to the right. Thus, regression path times are likely to reflect slightly later processes, such as integration of the critical region with the preceding context. The *percent regressions* indicate the probability that a reader made a regressive eye movement to preceding regions after fixating a given region. This measure includes only regressions made during the reader’s first pass through the region, and does not include regression made after re-fixating the region.

Reading time data (i.e., first fixation, first pass, and regression path durations) were analyzed using linear mixed effects models (Baayen et al., 2008), and percent regressions were analyzed by mixed effects logistic regression, as the dependent measure was categorical (see Jaeger, 2008). The mixed effects models included random intercepts for participants and items. We used the same procedure as Experiment 1 to simplify the random slope structure until the models converged in all regions and eye movement measures. This procedure led us to adopt verb transitivity as a random slope for participants and items for all fixation measures and regions, except for percent regression measures in the post-verb region. Here, we removed the verb transitivity random slope for participants, as the transitivity bias variance across different verbs (if any) is more likely to influence the data than variance in participants’ experience with the verbs.

When the critical region demonstrated a significant interaction of verb and structure type, a planned comparison was conducted with separate mixed effects models to test for systematic differences between the island and non-island conditions within each verb type. These models included participants and items as random intercepts.

##### Results

**Table 3** presents the participant means on each measure for each region as well as the standard errors of the participant means, and **Table 4** presents a summary of the statistical analyses.

**Table 3:** **Experiment 2 participant mean reading times in milliseconds (standard error) and percent regressions**.

Measure	Pre-verb region	Verb region	Post-verb region
**First fixation**
Transitive, non-island	212 (8)	249 (12)	242 (9)
Transitive, island	217 (7)	240 (7)	243 (9)
Intransitive, non-island	207 (8)	256 (10)	246 (10)
Intransitive, island	208 (5)	231 (8)	237 (7)
**First-pass time**
Transitive, non-island	287 (14)	277 (13)	283 (13)
Transitive, island	386 (20)	275 (10)	287 (12)
Intransitive, non-island	299 (15)	303 (13)	296 (14)
Intransitive, island	396 (19)	266 (11)	284 (10)
**Regression path time**
Transitive, non-island	463 (28)	373 (24)	402 (30)
Transitive, island	636 (43)	406 (31)	447 (35)
Intransitive, non-island	472 (38)	397 (23)	492 (35)
Intransitive, island	619 (41)	425 (38)	469 (26)
**Percent regressions**
Transitive, non-island	33.1 (5.0)	17.1 (3.5)	17.9 (3.4)
Transitive, island	33.2 (4.0)	23.0 (4.4)	24.4 (3.4)
Intransitive, non-island	27.1 (4.8)	16.2 (2.8)	27.5 (3.7)
Intransitive, island	32.7 (4.7)	24.4 (3.7)	22.4 (3.1)

**Table 4:** **Summary of model estimates, standard errors, and *t*-values (for linear mixed effects models) and z-values (for logit mixed effects models) for four eye movement measures in Experiment 2**.

Measure	Pre-verb region		Verb region		Post-verb region	
	Estimate	*t* (*Z*)	Estimate	*t* (*Z*)	Estimate	*t* (*Z*)
**First fixation**
(Intercept)	210 (6)	37.361	242 (6)	38.800	241 (7)	33.526
Verb	-6 (6)	-1.71	2 (8)	0.279	3 (6)	-0.418
Structure	3 (5)	0.559	15 (6)	-2.425^∗^	5 (6)	-0.780
Verb ^∗^ Structure	-1 (10)	-0.141	24 (13)	-1.887^†^	6 (12)	-0.529
**First-pass time**
(Intercept)	344 (14)	24.386	279 (8)	35.233	288 (11)	25.819
Verb	12 (17)	0.747	8 (12)	0.696	2 (9)	0.186
Structure	97 (13)	7.402^∗∗^	-18 (9)	-2.076^∗^	-2 (9)	-0.227
Verb ^∗^ Structure	-0.8 (26)	-0.031	-45 (17)	-2.562^∗^	-12 (18)	-0.655
**Regression path time**
(Intercept)	551 (33)	16.495	398 (23)	17.29	458 (26)	17.793
Verb	2 (28)	0.086	12 (30)	0.414	53 (37)	1.420
Structure	162 (27)	5.930^∗∗^	27 (25)	1.092	17 (30)	0.579
Verb ^∗^ Structure	-41 (55)	-0.750	-12 (49)	-0.238	-53 (60)	-0.880
**Percent regressions**
(Intercept)	-0.96 (0.21)	-4.350	-1.63 (0.20)	-8.192	-1.36 (0.18)	-7.672
Verb	-0.18 (0.17)	-0.919	0.05 (0.25)	0.202	0.20 (0.19)	1.060
Structure	0.18 (0.17)	1.057	0.44 (0.20)	2.182^∗^	0.06 (0.18)	0.344
Verb ^∗^ Structure	0.29 (0.34)	0.854	0.28 (0.40)	0.693	-0.66 (0.36)	-1.835^†^

*Verb = verb transitivity (transitive vs. intransitive); Structure = island type (non-island vs. island).*

*^†^p< 0.10; ^∗^p*< 0.05; ^∗∗^p< 0.001.

In the pre-verb region, the first pass time and regression path measures showed a main effect of structure (*p* < 0.001), with longer reading times in the island conditions than in the non-island conditions. This effect was expected because the pre-verb region in the island conditions contained the extra word *who*, which made it more likely to attract multiple fixations in that region. No other significant effects were observed in this region.

In the verb region, evidence for the hyper-active gap filling hypothesis was found in first fixation durations as well as in first pass measures. Both measures showed a main effect of structure with longer reading times for non-island conditions (*p*s < 0.05). First fixation durations showed a marginal interaction of structure and verb transitivity (*p* = 0.06), and first pass times showed a significant interaction (*p* < 0.05). Planned pairwise comparisons on first fixation durations and first pass times revealed that reading times in the non-island, intransitive condition were significantly longer than in the island, intransitive condition (*p*s < 0.01), but no significant difference was observed between the transitive conditions. No significant effect was observed for the regression path durations. There was a main effect of structure in percent regressions (*p* < 0.05), with a higher percentage of regression in the island conditions, which likely reflected the greater structural complexity in the island conditions.

In the post-verb region, there was a marginally significant interaction of verb and structure type (*p* = 0.066), but no significant effect was observed in other eye-movement measures.

##### Discussion

Experiment 2 used an eye-tracking during reading method to investigate whether the parser uses verb transitivity information in deciding whether to postulate a gap at the verb object position. First fixation durations and first pass times for intransitive verbs were significantly longer in a structure that allows a gap (non-island condition) than when the same verb appeared in an island configuration. This effect was not observed when the critical verb was transitive. The fact that there was a reading disruption for intransitive verbs but not for transitive verbs is consistent with the prediction of the hyper-active gap filling hypothesis. If the parser creates an object gap and integrates the filler into the object position before having access to verb transitivity information, reading disruption in the non-island intransitive condition should result from the mismatch between the predicted transitivity and actual transitivity of the verb.

It is also important to note that in this experiment the critical mismatch effects were observed in the verb region, unlike in Experiment 1 where the mismatch effects were observed only in the spill-over adverb region. This constitutes stronger evidence for hyper-active gap filling, because the mismatch effect must have resulted from properties of the verb itself. The question of why the critical effects were observed in the verb region in Experiment 2 (unlike in Experiment 1, where the effect was found in the spill-over region) likely reflects task-based differences whose effects are seen well beyond the current studies. Inhibition of the button pressing action in self-paced reading tasks is likely more difficult than inhibition of saccades in an eye-tracking task.

We note that one other methodological difference between our experiments and Staub (2007) regards the types of intransitive verbs used. Our intransitive materials consisted of two types of intransitive verbs: we mainly used unergative verbs which only take a semantic agent as an argument, but we also used unaccusative intransitive verbs that only take a theme/experiencer as an argument (Perlmutter, 1978; Levin and Rappaport Hovav, 1995). On the other hand, Staub’s intransitive condition used only unaccusative intransitive verbs. Both types of intransitive verbs are generally incompatible with an overt direct object NP, but in some restricted contexts unergative intransitive verbs are capable of hosting an NP object (e.g., “laugh a big laugh”; see Keyser and Roeper, 1984). It is possible that this special property of unergative verbs may have led the parser to treat it in the same way as transitive verbs in our experiments, whereas unaccusative intransitive verbs admit no such exceptions.

It is important to note that this difference in materials design does not challenge our interpretation of the data. First, our stimuli did not meet the lexical or structural condition for allowing unergative verbs to behave as transitive verbs. Second, if our participants treated the unergative verbs as transitive verbs, then there should have been no reason to observe a slow-down in the intransitive, non-island condition, contrary to the findings in Experiments 1 and 2. However, in order to ascertain that our findings are not restricted to unergative intransitive verbs, we conducted Experiment 3 in which we used only the unaccusative intransitive verbs that were used in Staub (2007).

### Experiment 3

The goal of Experiment 3 was to replicate the findings from Experiments 1 and 2 with a different set of intransitive verbs. We constructed new sets of stimuli that used only the unaccusative intransitive verbs used in Staub (2007). Given that unaccusative intransitive verbs are syntactically incapable of hosting an overt direct object NP, this class of intransitive verbs provides a stronger test of the transitivity mismatch effect.

#### Method

##### Participants

We recruited 44 native speakers of American English from the University of Maryland community. All had normal or corrected-to-normal vision, and were naïve to the purpose of the experiment. They received course credit or were paid $10 for their participation, which lasted around 40 min.

##### Materials

We created 24 sets of four sentences. The experimental design in this study is identical to that of Experiment 2 (see **Table 2**), except that the sentences were modified such that the critical verbs in all items were unaccusative intransitive verbs used in Staub (2007). These verbs included *remain, depart, prevail, emerge, arise, die, persist, disappear, erupt, appear, vanish, arrive*. According to Staub (2007), these verbs are considered to disallow transitive frames. Although it may be possible to find some rare counter-examples, we note that this should only work against the hyper-active gap filling hypothesis, because the possibility of transitive frame would eliminate reasons to observe a reading time slow-down. Thus, finding a robust mismatch effect on the intransitive verb region should eliminate any concerns about the potential transitivity of the intransitive verbs.

The 24 sentence sets were counter-balanced across four lists, such that each participant saw only one version of each of the target sentences. We used 12 intransitive verbs from Staub (2007), such that 2 of the 24 items used the same verb with a different context. Participants saw each intransitive verb twice across the course of the experiment, once in an island context and once in a non-island context. The target sentences were combined with 108 fillers of similar length and complexity.

##### Procedure

An SR Research (Mississauga, ON, Canada) Eyelink 1000 eye-tracker at the University of Maryland was used to record eye movements. The basic configuration of this eye-tracker as well as the instruction for participants was the same as for Experiment 2, except that the stimuli were displayed on a 17-inch monitor, which allowed a maximum of 100 characters per line. The entire experiment lasted ∼40 min. The experiment protocol for this study was approved by the Institutional Review Board at the University of Maryland.

##### Data Analysis

The data analysis procedure was the same as that of Experiment 2. The mixed effects models included random intercepts for participants and items. We used the same procedure as Experiment 2 to simplify the random slope structure until the models converged in all regions and eye movement measures. This procedure led us to adopt verb transitivity as a random slope for participants only.

##### Results

Mean comprehension accuracy for the experimental items was 91.9% across the four conditions, and did not differ across the four conditions. **Table 5** presents the participant means on each measure for each region as well as the standard errors of the participant means, and **Table 6** presents a summary of the statistical analyses.

**Table 5:** **Experiment 3 participant mean reading times in milliseconds (standard error) and percent regressions**.

Measure	Pre-verb region	Verb region	Post-verb region
**First fixation**
Transitive, non-island	229 (8)	277 (8)	268 (11)
Transitive, island	237 (8)	266 (8)	258 (9)
Intransitive, non-island	226 (7)	299 (10)	271 (9)
Intransitive, island	222 (6)	270 (9)	260 (9)
**First-pass time**
Transitive, non-island	367 (22)	319 (12)	330 (21)
Transitive, island	468 (29)	316 (14)	321 (16)
Intransitive, non-island	349 (19)	379 (13)	340 (15)
Intransitive, island	461 (21)	345 (20)	308 (14)
**Regression path time**
Transitive, non-island	529 (29)	386 (20)	553 (79)
Transitive, island	706 (47)	520 (44)	529 (45)
Intransitive, non-island	538 (43)	528 (38)	545 (40)
Intransitive, island	762 (48)	527 (54)	497 (43)
**Percent regressions**
Transitive, non-island	31.0 (3.7)	11.7 (2.7)	26.4 (3.8)
Transitive, island	26.3 (4.1)	28.4 (3.6)	25.9 (2.9)
Intransitive, non-island	26.7 (3.9)	14.4 (2.3)	24.0 (3.5)
Intransitive, island	32.1 (3.6)	24.0 (3.3)	21.2 (3.1)

**Table 6:** **Summary of model estimates, standard errors, and *t*-values (for linear mixed effects models) and z-values (for logit mixed effects models) for four eye movement measures in Experiment 3**.

Measure	Pre-verb region		Verb region		Post-verb region	
	Estimate	*t* (*Z*)	Estimate	*t* (*Z*)	Estimate	*t* (*Z*)
First fixation (Intercept)	229 (5)	45.584	279 (6)	47.583	264 (8)	35.193
Verb	6 (6)	1.005	8 (8)	0.974	2 (8)	0.295
Structure	3 (6)	0.456	-20 (8)	-2.504^∗^	-10 (7)	-1.436
Verb ^∗^ Structure	19 (12)	1.561	-21 (16)	-1.327	-8 (14)	-0.593
First-pass time (Intercept)	411 (21)	19.502	341 (10)	35.383	324 (15)	21.715
Verb	-5 (17)	-0.269	17 (12)	1.400	12 (12)	1.015
Structure	103 (17)	6.046^∗∗∗^	-23 (12)	-1.896^†^	-18 (12)	-1.551
Verb ^∗^ Structure	31 (34)	0.906	-78 (24)	-3.240^∗^	-1 (23)	0.052
Regression path time (Intercept)	663 (44)	14.345	491 (26)	18.914	527 (42)	12.408
Verb	-29 (37)	-0.782	71 (38)	1.869^†^	28 (50)	0.573
Structure	188 (29)	6.413^∗∗∗^	64 (32)	1.978^∗^	-20 (37)	-0.545
Verb ^∗^ Structure	-68 (59)	-1.167	-129 (65)	-2.002^∗^	-4 (73)	-0.058
Percent regressions (Intercept)	-1.02 (0.15)	-6.935	-1.56 (0.15)	-10.328	-1.25 (0.15)	-8.300
Verb	-0.39 (0.19)	-2.074^∗^	0.28 (0.22)	1.301	0.19 (0.20)	0.998
Structure	0.06 (0.16)	0.374	0.93 (0.20)	4.668^∗∗∗^	-0.09 (0.17)	-0.517
Verb ^∗^ Structure	-0.17 (0.33)	-0.533	-0.04 (0.40)	-0.088	0.33 (0.35)	0.959

*Verb = verb transitivity (transitive vs. intransitive); Structure = island type (non-island vs. island)*.

*^†^p< 0.10; ^∗^p< 0.05; ^∗∗∗^p< 0.001.*

Overall, the statistical analysis revealed a similar pattern to the results of Experiment 2. In the pre-verb region, first pass and regression path times showed a main effect of structure type (*p*s < 0.001), with longer reading times in the island conditions than in the non-island conditions. As explained above, this effect was expected since the pre-verb region in the island conditions contained the extra word *who*. Percent regressions showed a main effect of verb type (*p* < 0.05), with more frequent regressions in the intransitive conditions. Although this was unexpected, the regression frequency in the pre-verb region is unlikely to have affected reading times in subsequent regions.

In the verb region, first fixation durations revealed a main effect of structure type (*p* < 0.05), with longer reading times in the intransitive conditions, but the interaction was not significant. In first pass times, however, a significant interaction of verb and structure type effect was observed (*p* < 0.05). A pairwise comparison revealed that reading times in the non-island, intransitive condition were longer than in the island, intransitive condition (*p* < 0.001), but no significant difference was observed between the transitive conditions.

Because the regression path duration measure reflects differences in the probability of regressing from this region, we discuss the percent regressions results at the verb region first. There was a main effect of structure in percent regressions (*p* < 0.05). The greater percent regression in the island conditions most likely reflects the structural complexity of the island conditions. Next, regression path durations also revealed a main effect of structure (*p* < 0.05), as well as a significant interaction of verb and structure (*p* < 0.05). Pairwise comparisons revealed that the direction of this effect was the opposite of the expected pattern: a significant difference between the transitive conditions (*p* < 0.01), but no difference between the intransitive conditions.

This interaction was unexpected, but it receives a straightforward explanation once we consider the fact that regression path times reflects two different underlying measures: the first pass time and time spent regressing to earlier regions (for discussion see Staub and Clifton, 2006). As described above, transitivity mismatch was associated with longer first pass times and increased regressions in the intransitive non-island condition. However, the presence of an island appeared to have an independent cost as evidenced by the fact that the two island conditions had high percentages of regressions (24.0 and 28.4%), and this is reflected in the large regression path time in these conditions. In other words, the interaction in regression path may have resulted from the combination of complexity slowdowns in the two island conditions and transitivity mismatch slowdown in the intransitive non-island condition, such that all three were slower than the transitive non-island condition.

In the post-verb region, no significant effect was observed in any of the eye-movement measures.

##### Discussion

Experiment 3 was designed to replicate the results of Experiment 2 with the same intransitive verbs used by Staub (2007). We again observed that first pass times for intransitive verbs in a structure that would allow a gap (non-island condition) were significantly longer than when the same verb appeared within an island configuration. This contrast was not observed when the critical verb was transitive with a plausible direct object. This contrast is consistent with the hyper-active gap filling hypothesis, which states that the parser creates an object gap and integrates the filler into the object position before having access to verb transitivity information. This hypothesis predicts that reading disruption in the non-island intransitive condition should result from the mismatch between the predicted transitivity and actual transitivity of the verb.

We also found that regression path times at the verb region were much shorter for the transitive non-island condition than the other three conditions, a pattern that was also present but unreliable in Experiment 2. As discussed in the results section, this was due to a combination of the higher percentage of regressions in the island conditions and the longer first pass time in the intransitive non-island condition. Although speculative, one possible interpretation of the larger percentage of regressions in the island conditions is that island conditions contain an extra word (i.e., the relative pronoun *who*) and incur greater complexity.

### General Discussion

Experiments 1, 2, and 3 all demonstrated evidence for reading disruption at an intransitive verb when the verb was in a potential gap-filling environment. The reading disruption that can be attributed to a transitivity mismatch effect was observed in the same region as the plausibility mismatch effect (Experiment 1), and this reading disruption for an intransitive verb was observed as early as the first fixation on the intransitive verb (Experiments 2 and 3). These results lend support to the hyper-active gap filling hypothesis, which claims that in English filler-gap dependency processing, object gap creation can be initiated based on pre-verbal information and can thereby lead the parser to expect a transitive verb. This is indeed what has been proposed for filler-gap dependency processing mechanism in head-final languages (Aoshima et al., 2004), but the current work suggests that the same mechanism extends to the processing of filler-gap dependency in verb-medial languages like English as well.

The view that object gap creation is triggered by pre-verbal information contrasts with a standard view in English filler-gap dependency processing that object gap creation is driven by properties of the verb (e.g., Pickering and Barry, 1991; McElree et al., 2003). In fact, the hyper-active gap filling mechanism suggests an alternative interpretation of existing evidence for active object gap creation. For example, the plausibility mismatch effect found in Traxler and Pickering (1996) has been taken to suggest that filler-retrieval occurs after accessing the transitivity information on the verb, and that subsequent structural integration of the filler leads to the implausible verb–object composition, which in turn results in reading time slowdown. However, under the hyper-active gap filling account, prior to the verb the reader analyzes the filler as a direct object of the upcoming verb, and given the combination of the subject NP and the hypothesized object NP, the reader may already expect a certain class of transitive verbs that would be semantically compatible with the filler NP. In other words, plausibility mismatch effects could be reconsidered as a reflection of a violation of lexical expectations, which result from predictive structural analysis. Future studies are needed to examine to what extent this reinterpretation of plausibility mismatch effects is feasible.

The present study has focused on filler-gap dependency processing, but the current conclusion is consistent with a broader class of models of sentence processing that propose that the parser utilizes a variety of sources of linguistic and contextual information to predictively build structural representations (Kimball, 1975; Gibson, 1998; Hale, 2003; Kamide et al., 2003; Staub and Clifton, 2006; Levy, 2008). On the other hand, the present study does not reveal what kind of pre-verbal information is critical for triggering object gap creation in advance of the verb. One possible source that was already discussed in the Introduction is the grammatical knowledge of phrase structure rules, which suggest that the upcoming VP representation can contain an object NP slot. However, it is equally feasible that the parser could use non-grammatical information in predictively positing the object gap, such as differences in the relative conditional probabilities derived from the lexical and contextual information from the combination of the filler NP and the subject. For example, even when a clause appears to resemble a gap structure like a relative clause, with a certain combination an adjunct gap may seem much more plausible than an object gap analysis (e.g., *the day that*… can continue as involving an adjunct gap as in *the day that I was born,* or an object gap as in *the day that I have been looking forward to*). Further studies are needed to investigate what kind of information contributes to such predictive object gap creation processes (Chow et al., 2013).

We acknowledge that the data reported in this paper are compatible with an alternative explanation that assumes that verb information plays a critical enabling role in English filler-gap dependency formation. For example, it is possible that filler retrieval processes are automatically activated as soon as the parser accesses the category information of the verb without accessing the transitivity information of the verb. Such a procedure could be motivated by an incremental interpretation strategy that attempts to combine any N-N-V sequence into a proposition (for discussion, see e.g., Goodluck et al., 1991, 1995). Under this alternative account, the transitivity mismatch effect arises because the filler that was ‘blindly’ retrieved based on the verb category information mismatches the subcategorization property of the verb that is accessed later (see van Gompel and Liversedge, 2003, for a similar proposal for a gender mismatch effect in pronoun processing, and see Kazanina et al., 2007 for an alternative account based on predictive mechanisms).

Although our study does not completely rule out a non-predictive account, these data place important constraints on the form that such an account must take. Critically, a non-predictive account must assume that access to the contents of lexical information is ordered, such that category information is accessed earlier than the subcategorization property of the verb. However, as yet there is little evidence to support such ordered access to category vs. other contents of a verb (Farmer et al., 2006 is one rare case, but see Staub et al., 2009 for a counterargument), whereas there is an abundance of psycholinguistic and neurolinguistic research demonstrating extremely fast access to all aspects of lexical content (e.g., Federmeier et al., 2000; Dambacher et al., 2006; Hauk et al., 2006; Staub and Rayner, 2007; Tanenhaus, 2007; Almeida and Poeppel, 2013; Chow et al., 2014). Moreover, there has been a recent surge of empirical work demonstrating that structure building processes can proceed predictively based on various types of top–down linguistic and contextual information, as discussed above (e.g., Konieczny, 2000; Kamide et al., 2003; DeLong et al., 2005; Van Berkum et al., 2005; Lau et al., 2006; Staub and Clifton, 2006; Levy and Keller, 2013; Yoshida et al., 2013; Yoshida, unpublished doctoral dissertation), including access to transitivity information (Arai and Keller, 2013). The current work demonstrating extremely early object gap creation processes can be seen as another instance of such predictive structure building processes. While these other findings lead us to favor a predictive explanation, further work is needed to more firmly establish that the hyper-active gap filling hypothesis is a better account for the pattern of results observed across a variety of paradigms than this alternative category-driven approach.

The current finding may also seem to contradict findings by Boland et al. (1995) and Pickering and Traxler (2001). These authors tested the processing of filler-gap dependencies in sentences that contain verbs like *persuade* or *remind* that can have both an NP direct object slot and a clausal complement slot in their argument structure, and found no evidence for reading disruption when the filler was semantically incompatible with the direct object NP slot, but compatible with the complement slot. According to the hyper-active gap filling account, encountering a *persuade*-type verb should not result in a transitivity mismatch effect since *persuade* makes available an object position, but one may wonder whether it should result in a plausibility mismatch effect when the filler is a semantically incompatible object, since an object-gap structure is hypothesized to be predictively constructed before the verb.

We can see two ways of reconciling these findings with the results presented here. First, the plausibility mismatch slowdown observed for simple transitive verbs may largely reflect the cost of reanalyzing the predicted structure to one that is compatible with the new input, which may vary depending on the argument structure of the verb. Revision may be costly in the cases where the verb is intransitive or mono-transitive, where the argument structure does not provide sufficient information for the parser to anticipate an alternative structural position for the filler. In the *persuade/remind* cases, on the other hand, the revision may be less costly because the argument structure of the verb clearly indicates the presence of an upcoming clause in which the filler can be integrated. Second, the predicted filler-gap structure may be more abstract than we have indicated so far. Rather than specifically predicting an object gap when the filler and relative clause subject are encountered, the parser may simply predict an argument gap position somewhere inside the complement domain of an upcoming VP representation, such that a gap in either the NP slot or in the clausal complement slot of *persuade*-class ditransitive verbs would be consistent with the prediction. The current results are compatible with either account. In sum, under either account, reading disruption at the verb can be mitigated when the verb makes more than one argument position available. This might explain why having an adjunct PP continuation (e.g., about) for mono-transitive verbs (e.g., wrote) still causes reading disruption at the verb while ditransitive verbs like *persuade/remind* do not lead to such reading disruption.

In the sentences used here, the intransitive structures are eventually resolved by the appearance of a preposition, which provides another structural position for the filler. Although this could be recognized as a possible reanalysis even at the verb position, this adjunct position is not specifically licensed by the input until the preposition is actually encountered (in contrast with the *persuade/remind* cases, in which the object position is available at the verb due to its argument structure information). One interesting question for further research is whether the difficulty of recovering from the simple transitive analysis is modulated by the frequency with which a particular intransitive verb co-occurs with a prepositional phrase that could host the filler. For example, many intransitive verbs can be combined with a prepositional complement to form a phrasal verb that takes the object of the preposition as an argument, e.g., *listen to the music*. If a particular intransitive verb occurs very frequently in a phrasal verb configuration, reanalysis to this structure in a filler-gap configuration might be less costly, even prior to the presentation of the preposition.

Finally, the conclusion that the same filler-gap dependency completion procedure is used across head-initial and head-final languages suggests that the parser’s structure building procedures, at least for filler-gap dependency completion, may not be qualitatively different across languages. However, future studies extending beyond Japanese and English are needed to test the robustness of this generalization. Moreover, predictive dependency formation processes are observed in domains other than filler-gap dependency processing (e.g., resolution of backward anaphora; Kazanina et al., 2007; Aoshima et al., 2009; Yoshida et al., 2013), but it is not yet known whether these other predictive structure building processes are also relatively constant across languages. Overall, we believe this line of cross-linguistic investigation has the potential to shed further light on fundamental questions about the relationship between linguistic representations and real-time processes for constructing those representations.

### Conclusion

The present study tested the hypothesis that predictive structure building processes underlie filler-gap dependency completion in English. In the presence of a filler-gap dependency, intransitive verbs consistently led to reading disruption, and this pattern was replicated in self-paced reading measures as well as in eye movement measures. These findings show that English speakers do not wait to check that the verb makes an object position available, and are consistent with the hypothesis that the parser postulates an object gap at least as soon as it encounters a filler phrase and a subject NP. We suggest that the parser uses pre-verbal information to predictively create rich syntactic representations regardless of word order differences across languages.

### Conflict of Interest Statement

The authors declare that the research was conducted in the absence of any commercial or financial relationships that could be construed as a potential conflict of interest.

## Footnotes

1 In this paper we use the ‘gap-filling’ or ‘gap-creation’ terminology in a theory-neutral way, as is typical in the psycholinguistic literature. This terminology should not be taken as indicating a commitment to representations that include gaps or traces; all of the processing theories we discuss here could be specified in terms of representations that do not include empty categories.
