# Supplementary material for: Hyper-active gap filling
Source: Front Psychol. 2015 Apr 10;6:384. doi: 10.3389/fpsyg.2015.00384 (PMC4392588; doi:10.3389/fpsyg.2015.00384)
Supplement: Supplementary file 1 [file Presentation_1.PDF]

Supplementary Material

Hyper-active gap filling

Akira Omaki[1]*, Ellen Lau[2], Imogen Davidson White[2], Myles Louis Dakan[2], Aaron Apple[1], Colin Phillips[2]

[1]Department of Cognitive Science, Johns Hopkins University, Baltimore, MD, USA
[2]Department of Linguistics, University of Maryland, College Park, MD, USA

*** Correspondence:** Akira Omaki, Department of Cognitive Science, Johns Hopkins University, 3400 N Charles Street, 237 Krieger, Baltimore, MD, 21218, USA
omaki@jhu.edu

1. Materials used in Experiment 1

1a/c. The house that the woman wrote/grinned slyly about was inspected by the board.
1b/d. The house that the woman who wrote/grinned slyly appreciated was inspected by the board.
Example comprehension question: Was it the board that inspected the house?

2a/c. The city that the author wrote/chatted regularly about was named for an explorer.
2b/d. The city that the author who wrote/chatted regularly saw was named for an explorer.

3a/c. The injury that the victim called/sighed repeatedly about was treated improperly by doctors.
3b/d. The injury that the victim who called/sighed repeatedly saw was treated improperly by doctors.

4a/c. The jewelry that the sheriff questioned/joked sharply about was recovered after the robbery.
4b/d. The jewelry that the sheriff who questioned/joked sharply watched was recovered after the robbery.

5a/c. The billboard that the girl threw/snorted angrily at was located near the beach.
5b/d. The billboard that the girl who threw/snorted angrily saw was located near the beach.

6a/c. The wall that the boy threw/grinned mischievously at was painted fire engine red.
6b/d. The wall that the boy who threw/grinned mischievously noticed was painted fire engine red.

7a/c. The artist that the architect designed/competed passionately with was nationally well known.
7b/d. The artist that the architect who designed/competed passionately admired was nationally well known.

8a/c. The design that the professor lectured/sighed resignedly about was discussed in the seminar.
8b/d. The design that the professor who lectured/sighed resignedly saw was discussed in the seminar.

9a/c. The equipment that the employee phoned/frowned disapprovingly about was mentioned by the President.
9b/d. The equipment that the employee who phoned/frowned disapprovingly saw was mentioned by the President.

10a/c. The drugs that the principal threatened/frowned sternly about were discussed during the meeting.
10b/d. The drugs that the principal who threatened/frowned sternly confiscated were discussed during the meeting.

11a/c. The bridge that the tourist read/napped peacefully under was photographed by the group.
11b/d. The bridge that the tourist who read/napped peacefully missed was photographed by the group.

12a/c. The clock that the collector read/smiled fondly about was found while shopping for antiques.
12b/d. The clock that the collector who read/smiled fondly discovered was found while shopping for antiques.

13a/c. The bacteria that the biologist instructed/struggled intensely about turned out to be highly productive.
13b/d. The bacteria that the biologist who instructed/struggled intensely studied turned out to be highly productive.

14a/c. The match that the athlete trained/struggled endlessly for was ended by the authorities.
14b/d. The match that the athlete who trained/struggled endlessly played was ended by the authorities.

15a/c. The manager that the custodian cleaned/smiled obediently for was ruined by a financial crisis.
15b/d. The manager that the custodian who cleaned/smiled obediently liked was ruined by a financial crisis.

16a/c. The recording that the instructor taught/beamed enthusiastically about was heard throughout the auditorium.
16b/d. The recording that the instructor who taught/beamed enthusiastically presented was heard throughout the auditorium.

17a/c. The princess that the clown sang/danced cheerfully with was adored by the media.
17b/d. The princess that the clown who sang/danced cheerfully admired was adored by the media.

18a/c. The accident that the governor scolded/yelled angrily about was seen on the news.
18b/d. The accident that the governor who scolded/yelled angrily witnessed was shown on the news.

19a/c. The theories that the teacher scolded/corresponded vigorously about were taught throughout the term.
19b/d. The theories that the teacher who scolded/corresponded vigorously criticized were taught throughout the term.

20a/c. The jobs that the instructor taught/sulked grudgingly about were all in food service.
20b/d. The jobs that the instructor who taught/sulked grudgingly knew were all in food service.

21a/c. The country that the man killed/prayed endlessly for was destroyed by Mongol military.
21b/d. The country that the man who killed/prayed endlessly hated was destroyed by Mongol military.

22a/c. The party that the designer dressed/laughed obnoxiously for was thought to be very important.
22b/d. The party that the designer who dressed/laughed obnoxiously enjoyed was thought to be very important.

23a/c. The poster that the manager paid/appeared unexpectedly for was sent to the office.
23b/d. The poster that the manager that paid/appeared unexpectedly saw was sent to the office.

24a/c. The magazine that the children fought/giggled persistently about could not be found anywhere.
24b/d. The magazine that the children who fought/giggled persistently read could not be found anywhere.

25a/c. The sculpture that the critics lectured/quarreled seriously about was seen in the park.
25b/d. The sculpture that the critics who lectured/quarreled seriously denounced was seen in the park.

26a/c. The money that the criminal kidnapped/chuckled cruelly for was missing during the investigation.
26b/d. The money that the criminal who kidnapped/chuckled cruelly despised was missing during the investigation.

27a/c. The game that the journalist bet/shrieked compulsively on was discussed at the pub.
27b/d. The game that the journalist who bet/shrieked compulsively watched was discussed at the pub.

28a/c. The client that the cook prepared/flirted skillfully for was disliked by the waiters.
28b/d. The client that the cook who prepared/flirted skillfully favored was disliked by the waiters.

2. Materials used in Experiment 2

1a/c. The house that the woman wrote/grinned slyly about was inspected by the board.
1b/d. The house that the woman who wrote/grinned slyly appreciated was inspected by the board.
Example comprehension question: Was it the board that inspected the house?

2a/c. The city that the author wrote/chatted regularly about was named for an explorer.
2b/d. The city that the author who wrote/chatted regularly saw was named for an explorer.

3a/c. The injury that the victim called/sighed repeatedly about was treated improperly by doctors.
3b/d. The injury that the victim who called/sighed repeatedly saw was treated improperly by doctors.

4a/c. The jewelry that the sheriff questioned/joked sharply about was recovered after the robbery.
4b/d. The jewelry that the sheriff who questioned/joked sharply watched was recovered after the robbery.

5a/c. The billboard that the girl threw/snorted angrily at was located near the beach.
5b/d. The billboard that the girl who threw/snorted angrily saw was located near the beach.

6a/c. The wall that the boy threw/grinned mischievously at was painted fire engine red.
6b/d. The wall that the boy who threw/grinned mischievously noticed was painted fire engine red.

7a/c. The artist that the architect designed/competed passionately with was nationally well known.
7b/d. The artist that the architect who designed/competed passionately admired was nationally well known.

8a/c. The design that the professor lectured/sighed resignedly about was discussed in the seminar.
8b/d. The design that the professor who lectured/sighed resignedly saw was discussed in the seminar.

9a/c. The equipment that the employee phoned/frowned disapprovingly about was mentioned by the President.
9b/d. The equipment that the employee who phoned/frowned disapprovingly saw was mentioned by the President.

10a/c. The drugs that the principal threatened/frowned sternly about were discussed during the meeting.
10b/d. The drugs that the principal who threatened/frowned sternly confiscated were discussed during the meeting.

11a/c. The bridge that the tourist read/napped peacefully under was photographed by the group.
11b/d. The bridge that the tourist who read/napped peacefully missed was photographed by the group.

12a/c. The clock that the collector read/smiled fondly about was found while shopping for antiques.
12b/d. The clock that the collector who read/smiled fondly discovered was found while shopping for antiques.

13a/c. The bacteria that the biologist instructed/struggled intensely about turned out to be highly productive.
13b/d. The bacteria that the biologist who instructed/struggled intensely studied turned out to be highly productive.

14a/c. The match that the athlete trained/struggled endlessly for was ended by the authorities.
14b/d. The match that the athlete who trained/struggled endlessly played was ended by the authorities.

15a/c. The manager that the custodian cleaned/smiled obediently for was ruined by a financial crisis.
15b/d. The manager that the custodian who cleaned/smiled obediently liked was ruined by a financial crisis.

16a/c. The recording that the instructor taught/beamed enthusiastically about was heard throughout the auditorium.
16b/d. The recording that the instructor who taught/beamed enthusiastically presented was heard

throughout the auditorium.

17a/c. The princess that the clown sang/danced cheerfully with was adored by the media.
17b/d. The princess that the clown who sang/danced cheerfully admired was adored by the media.

18a/c. The accident that the governor scolded/yelled angrily about was seen on the news.
18b/d. The accident that the governor who scolded/yelled angrily witnessed was shown on the news.

19a/c. The theories that the teacher scolded/corresponded vigorously about were taught throughout the term.
19b/d. The theories that the teacher who scolded/corresponded vigorously criticized were taught throughout the term.

20a/c. The jobs that the instructor taught/sulked grudgingly about were all in food service.
20b/d. The jobs that the instructor who taught/sulked grudgingly knew were all in food service.

21a/c. The country that the man killed/prayed endlessly for was destroyed by Mongol military.
21b/d. The country that the man who killed/prayed endlessly hated was destroyed by Mongol military.

22a/c. The party that the designer dressed/laughed obnoxiously for was thought to be very important.
22b/d. The party that the designer who dressed/laughed obnoxiously enjoyed was thought to be very important.

23a/c. The poster that the manager paid/appeared unexpectedly for was sent to the office.
23b/d. The poster that the manager that paid/appeared unexpectedly saw was sent to the office.

24a/c. The magazine that the children fought/giggled persistently about could not be found anywhere.
24b/d. The magazine that the children who fought/giggled persistently read could not be found anywhere.

25a/c. The sculpture that the critics lectured/quarreled seriously about was seen in the park.
25b/d. The sculpture that the critics who lectured/quarreled seriously denounced was seen in the park.

26a/c. The money that the criminal kidnapped/chuckled cruelly for was missing during the investigation.
26b/d. The money that the criminal who kidnapped/chuckled cruelly despised was missing during the investigation.

27a/c. The game that the journalist bet/shrieked compulsively on was discussed at the pub.
27b/d. The game that the journalist who bet/shrieked compulsively watched was discussed at the pub.

28a/c. The client that the cook prepared/flirted skillfully for was disliked by the waiters.
28b/d. The client that the cook who prepared/flirted skillfully favored was disliked by the waiters.

3. Materials used in Experiment 3

1a/c. The studio that the students designed/remained peacefully in while the professors conferred was small and ugly.
1b/d. The studio that the students who designed/remained peacefully rested in while the professors conferred was small and ugly.
Example comprehension question: Was the studio ugly?

2a/c. The warehouse that the trucker phoned/departed nervously from last week was very old.
2b/d. The warehouse that the trucker who phoned/departed nervously visited last week was very old.

3a/c. The opponent that the veteran tennis player played/prevailed skillfully with/over during the game was very gracious.
3b/d. The opponent that the veteran tennis player who played/prevailed skillfully beat during the game was very gracious.

4a/c. The newsroom that the reporter called/emerged moodily from was full of problems.
4b/d. The newsroom that the reporter who called/emerged moodily oversaw was full of problems.

5a/c. The group that the speaker lectured/appeared reluctantly with at the conference was very dogmatic.
5b/d. The group that the speaker who lectured/appeared reluctantly spoke to at the conference was very dogmatic.

6a/c. The impaired plane that the pilot landed/arose wearily behind/from was a mass of twisted metal.
6b/d. The impaired plane that the pilot who landed/arose wearily had flown/crashed did not meet safety standards.

7a/c. The knight that the warrior killed/died nobly for went on to save the princess.
7b/d. The knight that the warrior who killed/died nobly admired/succumbed to went on to save the princess.

8a/c. The quarrel that the girl heard/persisted reluctantly about/in was incomprehensible and pointless.
8b/d. The quarrel that the girl who heard/persisted reluctantly resolved/won was incomprehensible and pointless.

9a/c. The building that the thief climbed/disappeared quickly over/behind during the chase was a nondescript warehouse.
9b/d. The building that the thief who climbed/disappeared quickly entered during the chase was a nondescript warehouse.

10a/c. The manager that the customer fought/erupted angrily with/at was near the register.
10b/d. The manager that the customer who fought/erupted angrily shouted at was near the register.

11a/c. The rave that the teenage boy planned/remained obligingly for/in at the underground club was hopping.
11b/d. The rave that the teenage boy who planned/remained obligingly watched at the underground club was hopping.

12 a/c. The old bed that the cat scratched/arose lazily at/in was really worn out but comfortable.
12b/d. The old bed that the cat who scratched/arose lazily sharpened her claws on was really worn out but comfortable.

13a/c. The table that the chemical burned/disappeared quietly on/from was very old and dirty.
13b/d. The table that the chemical that burned/disappeared quietly had left a stain on was very old and dirty.

14a/c. The people that the conqueror killed/prevailed fiercely for/over were [grateful to have their city back]/[upset to lose their city].
14b/d. The people that the conqueror who killed/prevailed fiercely defended were grateful to have their city back.

15a/c. The athlete that the coach taught/appeared proudly about/with before the game was nominated for a big award.
15b/d. The athlete that the coach who taught/appeared proudly trained before the game was nominated for a big award.

16a/c. The accident that the lady escaped/died mysteriously from/in last night was thoroughly investigated.
16b/d. The accident that the lady who escaped/died mysteriously had photographed last night was thoroughly investigated.

17a/c. The research that the scientist prepared/persisted determinedly for/in during his whole career was finally completed.
17b/d. The research that the scientist who prepared/persisted determinedly pursued during his whole career was finally completed.

18a/c. The gate that the limousine passed/emerged slowly through/from as it left the house was closed shortly thereafter.
18b/d. The gate that the limousine which passed/emerged slowly crossed as it left the house was closed shortly thereafter.

19a/c. The frat boy that the woman fought/erupted aggressively with/at in the department store was very obnoxious.
19b/d. The frat boy that the woman who fought/erupted aggressively disciplined in the department store was very obnoxious.

20a/c. The assistant that the magician trained/vanished skillfully with was good at her job.
20b/d. The assistant that the magician who trained/vanished skillfully complimented after the show was good at her job.

21a/c. The fugitive that the mobster hid/appeared abruptly from/with was rumored to be very dangerous.
21b/d. The fugitive that the mobster who hid/appeared abruptly feared/shot was rumored to be very dangerous.

22a/c. The airport that the ambassador left/departed rapidly for/from during the unrest was closed to most traffic.
22b/d. The airport that the ambassador who left/departed rapidly had visited during the unrest was closed to most traffic.

23a/c. The computer lab that the IT technician phoned/arrived tardily from/at was full of college kids studying for finals.
23b/d. The computer lab that the IT technician who phoned/arrived tardily despised was full of college kids studying for finals.

24a/c. The costume party that the student planned/arrived eagerly for at the fraternity house was pretty lame.
24b/d. The costume party that the student who planned/arrived eagerly attended/threw at the fraternity house was pretty lame.
